# Protective effect of (*E*)-(2,4-dihydroxy)-α-aminocinnamic acid, a hydroxy cinnamic acid derivative, in an ulcerative colitis model induced by TNBS

**DOI:** 10.1042/BSR20240797

**Published:** 2024-10-04

**Authors:** Astrid Mayleth Rivera Antonio, Itzia Irene Padilla Martínez, Yazmín Karina Márquez-Flores, Alan Hipólito Juárez Solano, Mónica A. Torres Ramos, Martha Cecilia Rosales Hernández

**Affiliations:** 1 Laboratorio de Biofísica y Biocatálisis, Sección de Estudios de Posgrado e Investigación, Escuela Superior de Medicina, Instituto Politécnico Nacional, Plan de San Luis y Salvador Díaz Mirón s/n, Casco de Santo Tomas, Ciudad de México 11340, México; 2 Laboratorio de Química Supramolecular y Nanociencias, Unidad Profesional Interdisciplinaria de Biotecnología, Instituto Politécnico Nacional, Avenida Acueducto s/n, Barrio la Laguna Ticomán, Ciudad de México 07340, México; 3 Departamento de Farmacia, Escuela Nacional de Ciencias Biológicas, Campus Zacatenco, Instituto Politécnico Nacional, Av. Wilfrido Massieu s/n Col. Zacatenco, C.P. 07738, Ciudad de México, México; 4 Dirección de investigación del Instituto Nacional de Neurología y Neurocirugía Manuel Velasco Suárez. Av. Insurgentes sur #3877, col. La Fama. Tlalpan, Ciudad de México. C.P. 14269. México

**Keywords:** hydroxycinnamic acids, inflammatory bowel disease, myeloperoxidase, oxidative stress

## Abstract

Ulcerative colitis (UC) is a multifactorial disease that causes long-lasting inflammation and ulcers in the digestive tract. UC is the most common form of inflammatory bowel disease (IBD). The current treatment for mild-to-moderate UC involves the use of 5-aminosalicylates (5-ASA), but much of this compound is unabsorbed and metabolized by N-acetylation. Several efforts have since been made to evaluate new molecules from synthetic or natural sources. Recently, it was reported that (*E*)-(5-chloro-2-hydroxy)-α-aminocinnamic acid (**2c**) and (*E*)-(2,4-dihydroxy)-**α**-aminocinnamic acid (**2f**) are as good or better myeloperoxidase (MPO) inhibitors and antioxidants than 5-ASA. Then, the present study aimed to evaluate the protective effects of **2c** and **2f** on a rat model of UC induced by 2,4,6-trinitrobenzene sulfonic acid (TNBS). The results showed that TNBS caused the induction of colonic ulcers, as well as a significant increase in MPO activity and malondialdehyde (MDA) and a decrease in glutathione (GSH) content. The administration of **2f, 2c** and 5-ASA, decreased the ulcers presence, inhibited MPO peroxidation activity and MPO presence (as determined by immunofluorescence), and increased GSH and reduced MDA content. However, **2f** was better than **2c** and 5-ASA, then, the principal mechanism by which **2f** presented a protective effect in a UC model induced by TNBS in rats is by inhibiting MPO activity and due to its antioxidant activity.

## Introduction

Ulcerative colitis (UC) and Crohn’s disease (CD) are types of inflammatory bowel disease (IBD), which is characterized by multifactorial and nonspecific processes [1]. In 2014 the overall incidence of UC was reported as 1.2 to 20.3 cases per 100,000 persons per year, with a prevalence of 7.6 to 245 cases per 100,000 persons per year [2]. In 2020, the incidence was 8.8 to 23.1 per 100,000 people per year in North America, 0.6 to 24.3 in Europe, and 7.3 to 17.4 in Oceania. In 2023 the prevalence of UC was around 5 millions of cases around the world [3]. However, there are few reports about the epidemiology of UC in Latin America [4]. Therefore, is important to observed that the incidence of UC had increasing over the years. UC occurs more frequently in individuals between 60 and 70 years of age. In addition, studies have shown that IBD is more common in urban areas [5] and affects men and women in equal proportions. However, symptoms in women are related to different factors, such as the menstrual cycle, fertility, and sexual health [6].

UC is a chronic inflammatory remitting disease that affects the colon; its pathogenesis is multifactorial and involves genetic predisposition, damage to the epithelial barrier, a dysregulated immune response, and environmental factors [7]. It is microscopically characterized by severe inflammation due to inflammatory cell infiltration, proinflammatory cytokine production, T cells and oxidative stress, and macroscopically characterized by the presence of diarrhea and rectal bleeding [8,9]. Oxidative stress could be a critical factor in the pathogenesis and perpetuation of mucosal damage in IBD, since it has been shown that neutrophils and monocytes in patients with active IBD produce higher concentrations of reactive oxygen species (ROS) [10] and myeloperoxidase (MPO) [11]. MPO is present in both neutrophils and macrophages, however, is more abundantly in neutrophils accounting for ∼5% of their dry mass (∼10 × 10^−6^ μg MPO/cell) and in macrophages is 60% less than in neutrophils [12]. Consequently, high levels of this enzyme and activity are commonly expected during UC because neutrophils are recruited to sites of inflammation. In addition, some studies have reported that MPO can be used as a biomarker in IBD due to its increased activity in the serum of patients with UC and CD [13].

High levels of MPO in neutrophils and macrophages contribute to damage to host tissue because MPO catalyzes hypochlorous acid (HOCl) production via the reaction between hydrogen peroxide (H_2_O_2_) and chloride (Cl^−^). HOCl degrades host tissue by activating protease and collagenolytic metalloproteinases, favoring protein-mediated degradation of the interstitial matrix of the mucosa and epithelial cells [14].

Therefore, to study UC, several *in vivo* murine chemically induced models have been reported, such as dextran sulfate sodium (DSS), acetic acid, and 2,4,6-trinitrobenzene sulfonic acid (TNBS) models; the latter is a hapten that binds to proteins in intestinal tissue and induces inflammatory responses. Studies have shown that TNBS-induced colitis comprises two forms of IBD, UC and CD, through the activation of Th1, Th2, and Th17 cell responses [15,16].

One of the pharmacological treatments for UC consists of 5-aminosalicylate (5-ASA), which is administered to more than 90% of patients within one year of diagnosis; in long-term follow-up, between 60% and 87% of patients continue to receive 5-ASA since it has been shown to protect against colorectal cancer [7,17–20]. 5-ASA protects epithelial barrier function in T84 cells against peroxynitrite (ONO_2_), which scavenges free radicals due to its antioxidant capacity [21]. Additionally, 5-ASA exhibits anti-inflammatory activity by inhibiting the synthesis of leukotrienes, prostaglandins, and proinflammatory cytokines [22] and inhibited MPO in studies *in vivo* and *ex vivo*. Also, by *in silico* studies interact in the active site of MPO and shows good affinity [23,24]. However, 5-ASA has a limitation that it is only used in mild to moderate cases of the disease [25] and present adverse reactions such allergies [26]. Also, thiopurine a inmmune-suppressant and anti-inflammatory agent is used. For the severe UC phase glucocorticoids or anti-tumor necrosis factor agent are employed [2]. However, glucocorticoids treatment for long periods are not recommended, and several of these treatments present several adverse reactions such as nausea, vomiting, several diarrhea, etc [27]. Then, despite to pharmacological treatment between 10 and 20% of patients could require proctocolectomy. Therefore, the natural products or their derivatives could be a good option during the UC pharmacological treatment.

Recent studies have shown that cinnamic acid and its derivatives such as caffeic acid and ferulic acid act by decreasing inflammatory pathways through the reduction of proinflammatory mediators such as TNF-α, IL-6, NF-κB, and MPO, the infiltration of immune cells and exhibited antioxidant action by maintaining redox regulation, in a model of ulcerative colitis, in addition to regulating the intestinal microbiota [28–31]. Furthermore, they suppressed the activation of bone marrow-derived macrophages due to their antioxidant properties [16,32]. Recently, a set of cinnamic acid derivatives (*E*)-2-Hydroxy-α-aminocinnamic acids was reported in which compounds **2c** and **2f** inhibit MPO activity at 100 μM, compound **2c** inhibit 40.5 ± 4.4% and compound **2f** 63.0 ± 1.5% in the peroxidation activity. And by 40.2 ± 1.2 (**2c**) and 29.6 ± 0.3% (**2f**) in the chlorination activity. Also, **2f** compound present a radical scavenging percentage near to 80 and 100% on DPPH and ABTS respectively. The cytotoxic potential of the compounds was evaluated at concentrations of 100 and 200 μM in the fibroblast cell line NIH/3T3 and was found that the cell viability with the **2c** and **2f** was higher that 80% [33,34]. Therefore, in this work, the anti-inflammatory, antioxidant, and MPO inhibitory activities of **2c** and **2f** were evaluated in an experimental ulcerative colitis model induced by the rectal administration of 2,4,6-trinitrobenzene sulfonic acid (TNBS) in Wistar female rats. Inflammation was determined by macroscopic and microscopic markers of damage, the disease activity index (DAI), and MPO activity and its presence in the mucosa, which indicated neutrophil infiltration. In addition, the antioxidant activities of **2c** and **2f** were also established by quantifying MDA and GSH.

The results showed that **2f** compound significantly reduced the presence of ulcers, inhibited MPO peroxidation activity and its presence in tissues, as assessed by immunofluorescence, increased GSH, and decreased MDA levels. In addition, Compound **2f** showed a better inhibitory effect than the reference compound (5-ASA) to act as an antioxidant and inhibiting MPO in a UC model, which could be due to the two hydroxyl groups in the aromatic ring which gives electron-donating properties and better resonance structures to maintain its stabilization.

## Materials and methods

### Chemicals

2,4,6-Trinitrobenzenesulfonic acid (TNBS), 5-aminosalicylic acid (5-ASA), ethanol, hydrogen peroxide, o-dianisidine hydrochloride, hexadecyltrimethyl ammonium bromide (HETAB), thiobarbituric acid (TBA), trichloroacetic acid (TCA), and ethylenediaminetetraacetic acid (EDTA) were obtained from Sigma‒Aldrich and used without further purification.

### Synthesis of compounds 2c and 2f

Compounds **2c** and **2f** were obtained as zwitterions following a microwave-assisted acid hydrolysis protocol with slight changes, as reported previously by Rivera Antonio et al. [34], from 3-acetamidocoumarins **1c** and **1f** and neutralization with NaHCO_3_ after hydrolysis. The reaction conditions were as follows: H_2_SO_4_ (15%) aqueous solution, temperature of 160°C for 20 min for **2c** and H_2_SO_4_ (5%) aqueous solution, temperature of 120°C for 10 min for **2f**.

IR spectra were recorded at 25°C with a Perkin Elmer Spectrum GX series with an FT system spectrophotometer using an ATR device. Melting points were measured in an Electrothermal IA 91000 device. ^1^H and ^13^C NMR spectra were acquired on a Varian Mercury NMR spectrometer operating at 300 MHz (1H, 300.08; 13C, 75.46 MHz) or Bruker Avance DPX-400 using DMSO-d6 as a solvent.

### Animals used for experimentation

Thirty-six female Wistar rats were used in the experiments. Even though the prevalence of UC is equal in males and females, the prevalence of symptoms is greater in females. Rats weighing approximately 250–300 g were used and kept under a light/dark (12 h) cycle at room temperature. The rats were fed a rodent diet (Lab Diet 5013) and water ad libitum. All rats were purchased from Facultad de Ciencias de la UNAM and maintained at the Laboratorio de Biofísica y Biocatálisis in Escuela Superior de Medicina del Instituto Politécnico Nacional. Animal procedures were carried out following the Technical Specifications for the Production, Care and Use of Laboratory Animals, SAGARPA, the ‘Guide for the Care and Use of Laboratory Animals’ of the National Research Council and the Official Mexican Standard. In addition, *in vivo* experiments were performed following the Animal Research: Reporting of In Vivo Experiments (ARRIVE) guidelines. The current protocol was approved by the Research Committee of the Escuela Nacional de Ciencias Biológicas (CEI-ENCB) (ENCB/CEI/038/2023).

### Induction and treatment of UC

The rats were randomly divided into six groups, with six animals in each group (*n*=6). The animals were fasted for 24 h before UC induction via the intrarectal administration of 2,4,6-trinitrobenzene sulfonic acid (TNBS) as follows. The animals were anesthetized with pentobarbital for insertion of a medical-grade polyurethane catheter (external diameter 2 mm) into the anus, and the tip was advanced to 8 cm proximal to the anus verge to administer 50 mg of TNBS (Sigma, St Louis, MO, U.S.A.) dissolved in 0.25 ml of a solution of ethanol: water (1:1). The animals were maintained in a head-down position for 2–3 min to prevent TNBS leakage. This administration was performed only once during the experiment [35,36]. After TNBS administration, on the same day, treatment with 5-ASA and the **2c** and **2f** compounds was started, and each group was named according to the treatment administered as follows:

**Sham**: without treatment
**EtOH** was administered rectally with a solution of ethanol:water (1:1) and orally with 0.4 ml of water and DMSO (0.2%), which were used to dissolve the compounds [37]
**TNBS**: TNBS + 0.4 ml of water and DMSO (0.2%) [38]
**5-ASA**: TNBS+ 5-ASA (50 mg/kg/day dissolved in 0.4 ml water and DMSO [0.2%]) [39]
**2c:** TNBS + **2c** (50 mg/kg/day dissolved in 0.4 ml of water and DMSO [0.2%])
**2f:** TNBS + **2f** (50 mg/kg/day in 0.4 ml of water and 0.2% DMSO)

All treatments were administered for 14 days.

### Assessment of disease activity index (DAI)

After 48 h of treatment with TNBS and each of the compounds, different clinical parameters were measured to determine whether UC was present because it has been reported that the disease occurs 48 h after induction. Body weight, stool consistency, and occult blood in the stool or anus were recorded. The amount of occult blood in the stool was determined using the Hema Screen manual rapid test on a card (Licon). The disease activity index (DAI) was scored based on the percentage of weight loss (0 ≤ 1%, 1 = 1–5%, 2 = 5–10%, 3 = 10–15%, 4≥15%), stool consistency (0 = normal, 2 = loose stools, 4 = diarrhea) and the presence/absence of blood in the stool (0 = negative, 2 = positive, 4 = gross bleeding) [11].

### Colonic sample preparation

Two weeks after UC induction and treatment, all the rats were euthanized using sodium pentobarbital (150 mg/kg) administered via intraperitoneal injection. The colonic tissue samples were obtained and divided into two parts of 10 cm each. One part was fixed in 4% paraformaldehyde to prepare tissue sections for microscopic examination and fluorescence. The other part of the tissue was stored at −80°C until biochemical evaluation.

### Macroscopic evaluation of colonic tissues

The macroscopic scoring system was used to assess the severity of colonic damage. Tissues from the sham group were scored for a normal appearance. Macroscopic considerations were scored as follows: (1 point) localized hyperemia without ulcer, (2 points) localized hyperemia with an ulcer, (3 points) a linear ulcer with inflammation at one site, (4 points) two or more ulcers with damage extending 1–2 cm along the length of the colon, and (5–8 points) damage that extends >2 cm along the length of the colon; the score increased by 1 for each cm suspected of involvement [11].

### Histology of colonic tissues

Colon samples were fixed in 4% paraformaldehyde in PBS at pH 7.4 for 12–18 h at room temperature.

### Paraffin embedding

The samples were subsequently dehydrated in a LUPETEC tissue processor (PT05TS), starting with an alcohol gradient of 60, 70, 80, 90, 96, 100 (1) and 100% (2). Then, the samples were passed through 50% v/v absolute alcohol supplemented with xylol, 100% xylol (1), 100% xylol (2), paraffin (Paraplast Surgipath Leica ref.39601006; 1), and paraffin (2). They were kept in each of the different solutions for 30 min. The transverse orientation of the sample was used to include it in paraffin, and the sample was allowed to cool to room temperature until the next day.

Cuts of 5 µm were made on a microtome (Reichert-Jung 1130/Biocut). Sections of the samples were loaded onto slides with 1% nutritive gelatin (BD Bioxon Nutritive gelatin) and stained with hematoxylin–eosin.

### Hematoxylin–eosin staining

For 40 min, the slides were kept at 58°C. Later, the sections were rehydrated as follows: 10 min in one xylol, 5 min in a second xylol, and 3 min in each of the subsequent alcohols under constant agitation (absolute alcohol, 96, 90, 80, 70, and 50%). Finally, the sections were rinsed in distilled water. Afterward, the samples were stained with Gill’s hematoxylin solution for 3 min and rinsed in distilled water for 3 min under constant agitation. Then, the slides were rinsed five times with 1% ammonium hydroxide and five times with distilled water, and they were kept for 5 min under constant agitation in alcohol at 50%. Then, the slides were stained with 1% eosin Y solution for one min. The sections were dehydrated in the following sequence of alcohols for 3 min each with constant agitation: 70, 80, 90%, and absolute alcohol. Then, the alcohol was extracted using two changes of xylene for five minutes each. Finally, we used mounting permanent medium (Entelan, Sigma-Aldrich) to cover the sections with a coverslip and left them dry at room temperature.

### MPO peroxidation activity

MPO peroxidation activity was quantified using the method described by Bradley with some modifications [40]. Colon samples were homogenized with 200 µl of phosphate buffer (50 mM/pH 6) supplemented with 0.5% hexadecyltrimethylammonium bromide (HTAB), 28 µl of supernatant and 172 µl of a mixture of hydrogen peroxide (30%) and o-dianisidine in phosphate buffer (PBS) at final concentrations of 0.0050% (1632 µM) and 167 µg/ml (684 µM), respectively, in a 96-well plate. The reaction mixture was incubated for 30 min at room temperature, and the absorbance was measured at 460 nm (Multiskan-EX Thermo Scientific, Thermo Fisher Scientific, Waltham, MA, U.S.A.). This assay was performed in triplicate [40].

### Determination of MPO by fluorescence microscopy

The colon samples were rinsed with PBS (pH 7.4), and 30% sucrose in PBS was added until the tissue sank (approximately 3–4 days). The sample was embedded in tissue tek (Sakura), and multiple floating sections were cut at 8, 20, 30, or 40 µm using a cryostat (Leica CM 1850, Germany). Finally, the sections were rinsed in PBS and stored at 4°C in a new PBS solution until immunostaining.

The identification of MPO was carried out in 40 µm float sections obtained from the rats of the different treatment groups (sham, EtOH, TNBS, 5-ASA, **2c** and **2f**) and preserved in paraformaldehyde at 4%.

The tissues were placed in 24-well plates, washed three times with 300 µl of PBS-Tween (PBST), and permeabilized with 300 µl of 2 mM pH:6 citrate buffer (permeabilizing solution) for 40 min at 70°C. At the end of this time, three washes were performed with 300 µl of 4% PBST, and the plates were blocked with 400 µl of a 1% BSA solution and 0.5% Tween in PBS (blocking solution) for 1 h at room temperature. After three washes were performed with 300 µl of PBST, the conjugated primary antibody (MPO polyclonal antibody, ALEXA FLOUR 350 conjugated, Bioss) was added, and the plates were diluted 1:200 in blocking solution and left overnight at 4°C. On the following day, three washes were performed with 300 µL of PBS in the dark, and the nuclei were stained for 5 min with propidium iodide (1:2000). Three washes with 300 µl of PBS were performed in a low-light room to eliminate the excess. Finally, the coverslips were carefully removed and placed on a slide with vectashield, and the coverslips were sealed with transparent nail varnish for observation under a BioTek Cytation 5 Cell Imaging Multimode Reader.

### Lipoperoxidation measurement

Malondialdehyde (MDA) was measured as a final product of the lipoperoxidation process based on the method described by Fevzi Polat et al. 2002 [41,42]. Fifty milligrams of colonic tissue from each rat was weighed. The samples were homogenized and subsequently centrifuged at 3904 × ***g*** for 15 min. Then, 150 μl of the supernatant and 100 μl of 150 mM Tris-HCl buffer (pH 7.4) were mixed and placed in a water bath at 37°C for 30 min. Then, 1 ml of thiobarbituric acid dissolved in trichloroacetic acid (TBA 0.3%/TCA 15%) was added to each tube, and the temperature of the water bath was increased to 100°C and incubated for 60 min. After the samples were centrifuged at 3904 × ***g*** for 15 min, 150 μl of the supernatant was added to a 96-well plate, and the absorbance was read at a wavelength of 540 nm in a Thermo Scientific™ Multiskan SkyMicroplate spectrophotometer. The assay was performed in triplicate.

### Measurement of reduced glutathione (GSH)

GSH measurements were carried out using Ellman’s reagent 5,5'-dithiobis-(2-nitrobenzoic acid) (DTNB) as described by Mourad et al. 2000, with slight modifications [43]. Fifty milligrams of colonic tissue from each rat was weighed and homogenized with 500 μl of 5% metaphosphoric acid and subsequently centrifuged at 3904 × ***g*** for 15 min. In an Eppendorf tube, 150 μl of the supernatant was mixed with 100 μl of 5% w/v metaphosphoric acid and 750 μl of 0.1 M PBS (pH 8). Finally, 15 μl of DTNB was added. Then, 150 μl of the mix was added to a 96-well plate, and the absorbance at a wavelength of 412 nm was read with a Thermo Scientific™ Multiskan SkyMicroplate spectrophotometer. The assay was performed in triplicate.

### Statistical analysis

The results are expressed as the mean ± SEM. One-way analysis of variance (ANOVA) with Tukey’s post-hoc test was performed to compare the data obtained between the TNBS and test groups. *P*<0.05 was considered to indicate a statistically significant difference [11].

## Results

### Chemical synthesis and characterization of Compounds 2c and 2f

**Figure 1:**
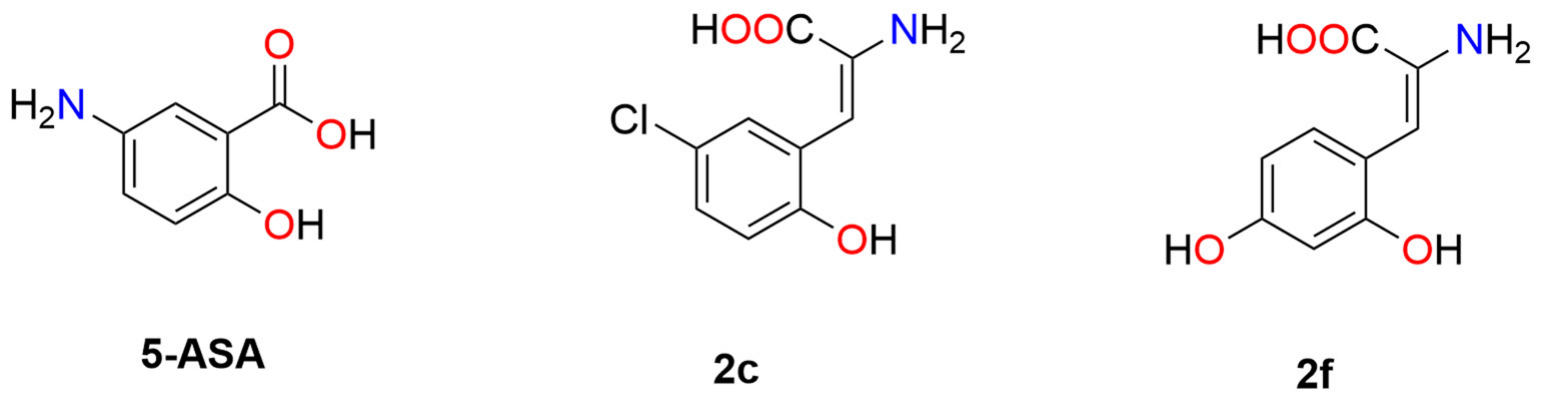
Chemical structures of **5-ASA and** (*E*)-2-hydroxy-α-aminocinnamic acids **2c** and **2f**

Cinnamic acid derivatives **2c** and **2f** (Figure 1) were obtained in moderate to good yields (63% and 67%, respectively) from the corresponding 3-acetamido coumarins **1c** and **1f**. Compounds **1c** and **1f** were synthesized following a reported method with modifications in terms of the quantities of the reagents used and reaction time, and starting from glycine instead of *N*-acetyl glycine, the characterization was performed according to the information reported by Rivera-Antonio et al. (2021), demonstrating the generation of Compounds **2c** and **2f** (shown in Supplementary Figures S1 and S2) [34].

### Disease activity index in UC

**Figure 2:**
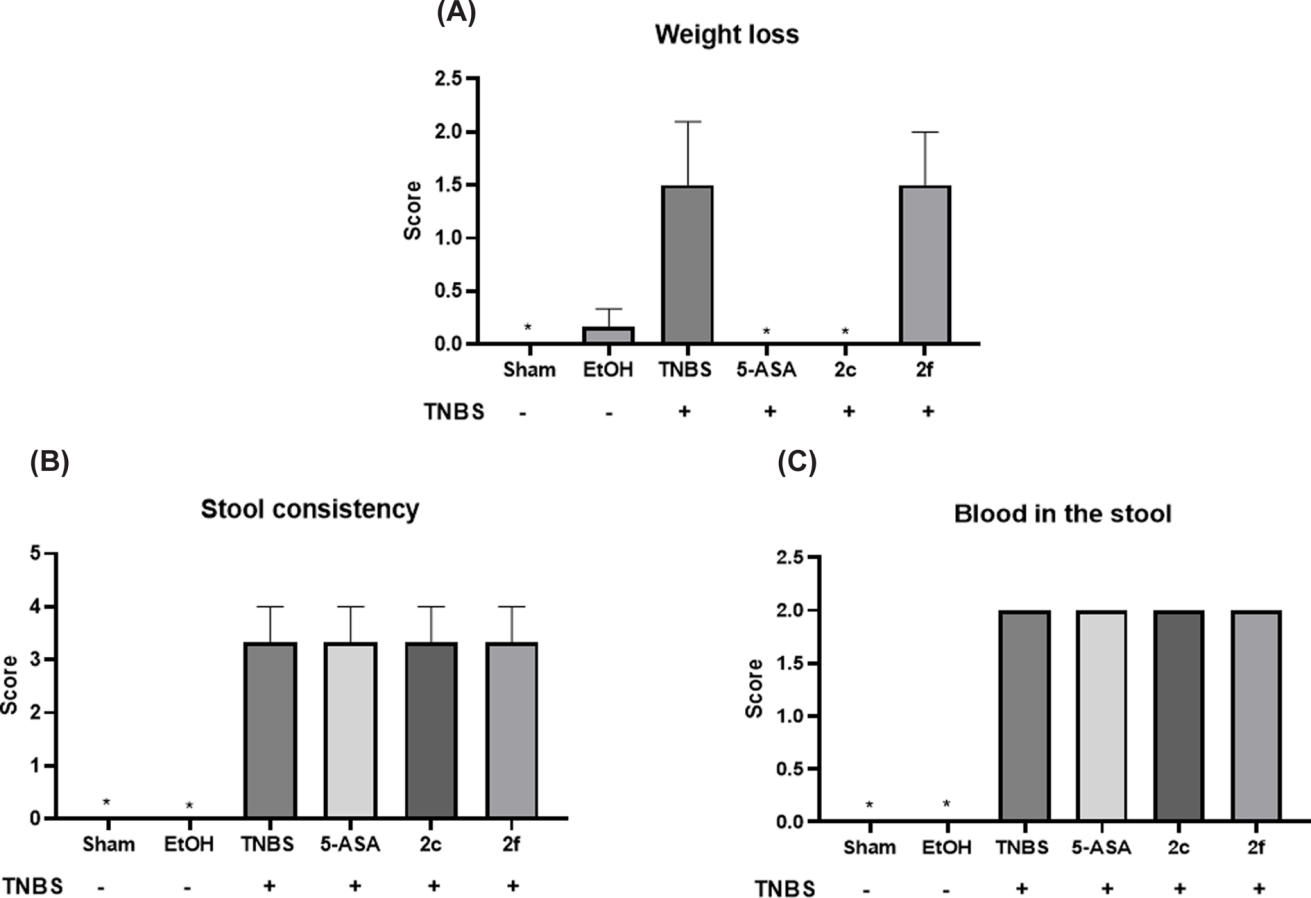
Disease activity index (DAI) of samples from the colon (**A**) Weight loss. (**B**) Stool consistency. (**C**) Blood in the stool. The values are the mean ±SEM, Sham; EtOH; TNBS; 5-ASA (50 mg/kg); **2c** (50 mg/kg), **2f** (50 mg/kg). *n* = 6 assay. Each measured was performed in triplicate. *P<0.05 indicates a statistically significant difference compared with the TNBS group.

The disease activity index (DAI) was calculated for each group after 48 h of UC induction with TNBS, considering weight loss (Figure 2A), stool consistency (Figure 2B), and the presence/absence of blood in the stool (Figure 2C). After the DAI was assigned, the DAI of the sham group was zero because the disease was not induced in this group. The EtOH group had a score of 0.16 because some animals in this group experienced weight loss, as shown in Figure 2A, but no blood was present in the stool (Figure 2C).

Compared with the sham and EtOH groups, the TNBS group had the highest DAI, which was equal to 6.83 ± 0.54 (*P*<0.001). The animals in the TNBS group exhibited weight loss (score of 1.5), diarrhea (score of 3.33), and the presence of occult blood in the stool (score of 2) (Figure 2A–C).

In the 5-ASA and **2c** groups, the DAI decreased compared with that in the induced group (TNBS), with a DAI of 5.33 ± 0.34. However, although the DAI decreased, the difference was not statistically significant compared with that in the TNBS group. Treatment with Compound **2f** did not affect the DAI after 48 h, with a value of 6.83 ± 0.39. Therefore, these results indicated that all groups in which TNBS was administered showed significant changes compared with those in the sham and EtOH groups, indicating that UC was present.

### Macroscopic evaluation of colonic tissue

**Figure 3:**
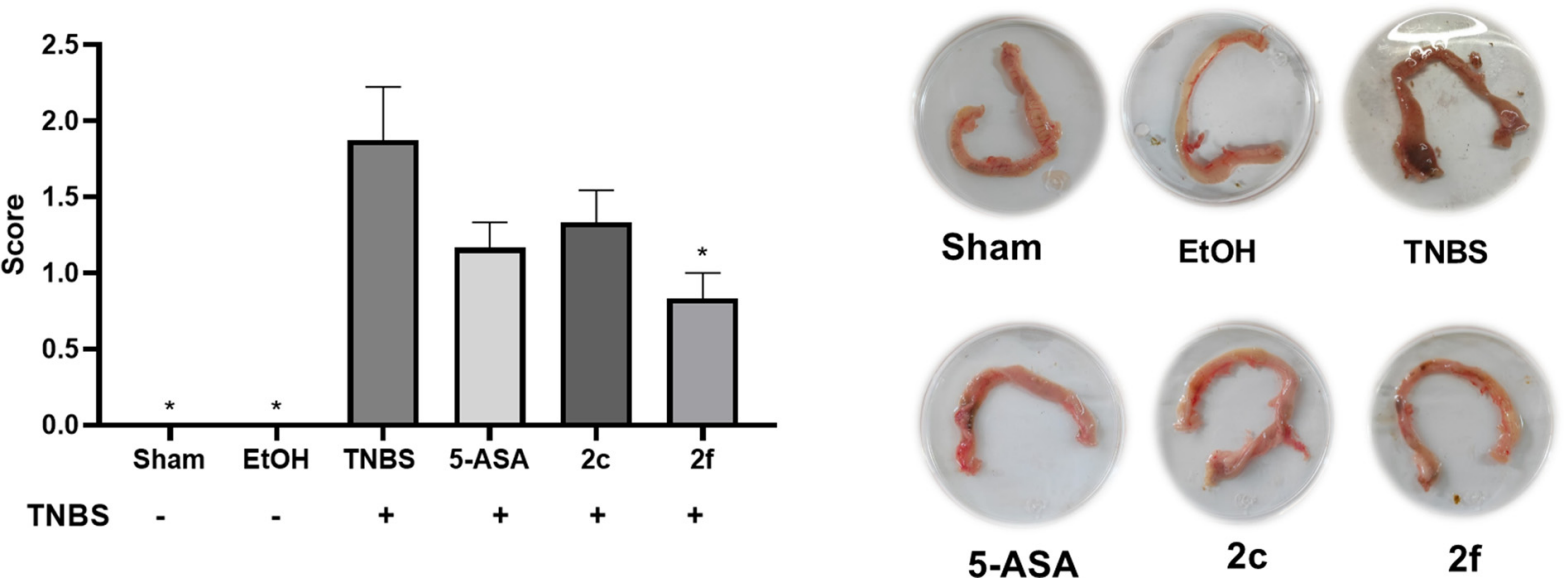
Colonic macroscopic damage score Macroscopic scores were used as a criterion to evaluate colon damage resulting from TNBS induction in the different groups: Sham; EtOH; TNBS; 5-ASA (50 mg/kg); **2c** (50 mg/kg); and **2f** (50 mg/kg). The data are presented as the means ± SEMs for six animals. *P<0.05 indicates a statistically significant difference compared with the TNBS group.

Once the colonic tissue samples were obtained, macroscopic evaluations were performed. Compounds **2c** and **2f** exhibited beneficial effects on the TNBS treatment. Macroscopic evaluation was carried out by observing the size and number of ulcers in the tissue and the presence of adhesions (S3). Compared with the group induced with TNBS, the group treated with Compound **2f** presented a greater reduction in macroscopic damage than did the 5-ASA group, as shown in Figure 3.

### Histology of the colonic tissue

**Figure 4:**
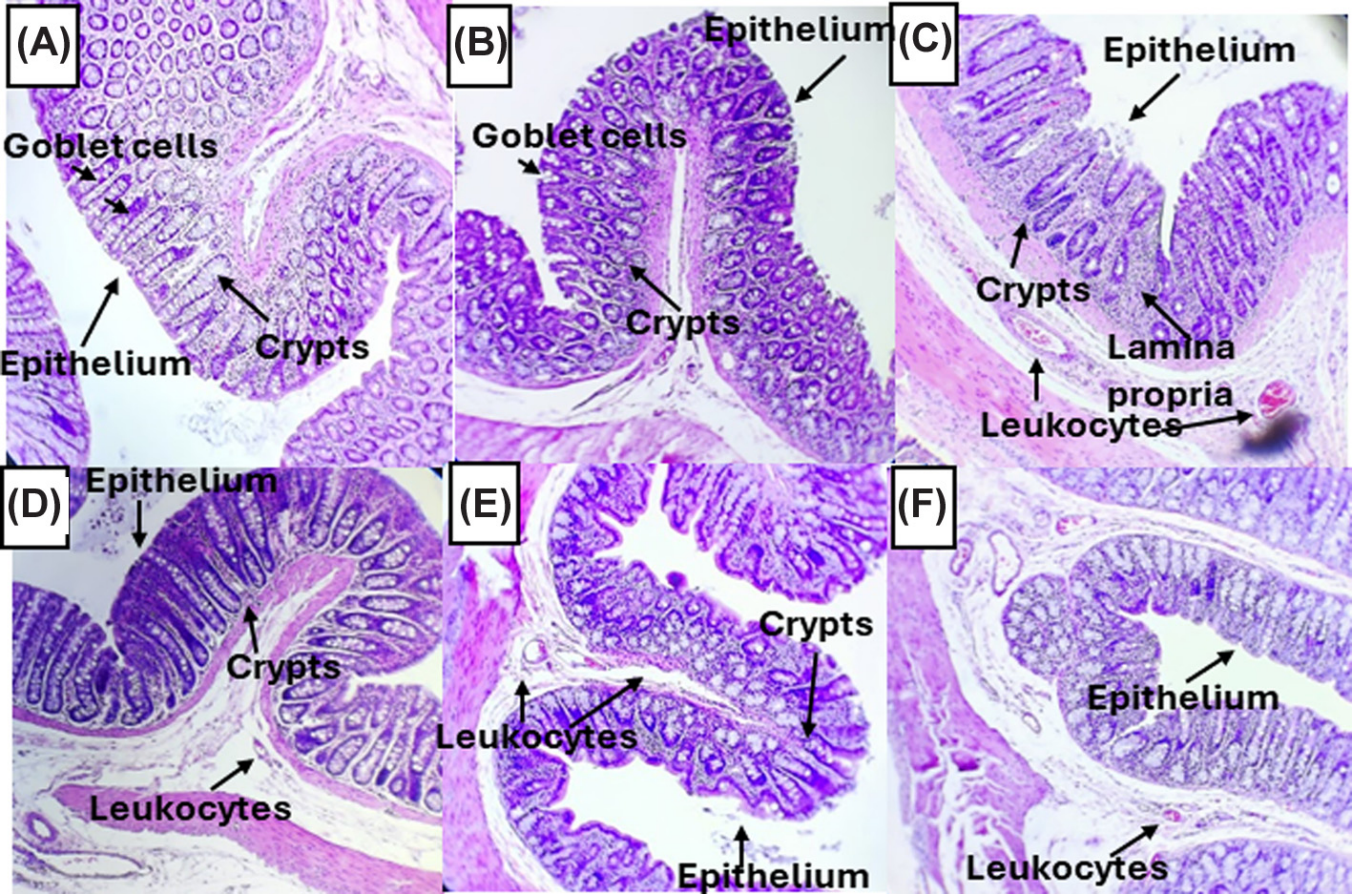
Colonic microscopic damage score Microscopic observation of colonic samples from (**A**) sham rats and those treated with (**B**) EtOH, (**C**) TNBS, (**D**) 5-ASA, (**E**) **2c** (50 mg/kg) or (**F**) **2f** (50 mg/kg) in a TNBS-induced UC model. Each sample from each rat was observed an representative image is shown. The images were taken at 10×.

A colonic tissue section from each rat was analyzed by histology. Figure 4 shows a representative image from each group, where no morphological changes were present in the Sham group (Figure 4A), indicating the absence of an inflammatory process. In the EtOH group, small clusters of leukocytes were observed between crypts (Figure 4B), whereas in the TNBS-induced group (Figure 4C), leukocytes were observed in the lamina propria due to infiltration in the superficial epithelium and hemorrhages between crypts, indicating inflammation caused by TNBS.

It is important to mention that EtOH group exhibited inflammation and oxidative stress because ethanol and its metabolite (acetaldehyde) have different mechanisms for disrupting the epithelial barrier, increasing the permeability of the intestine [37,44]. Although the morphology of the group treated with 5-ASA (Figure 4D) was better than that of the group treated with only TNBS, the presence of leukocytes in the lamina propria was observed. For the group treated with **2c** (Figure 4E), although the macroscopic damage in the tissue decreased, there was still leukocytes in the lamina propria.

In the image corresponding to the group induced with **2f** (Figure 4F), the epithelium was almost healed since it was less damaged, as was also observed in the 5-ASA group. Consistent with the macroscopic results, a decrease in ulcers and tissue damage was observed in the **2f** group compared with the 5-ASA group, although light signs of inflammation, such as hemorrhage in the lamina propria, were observed.

### Determination of MPO peroxidation activity

**Figure 5:**
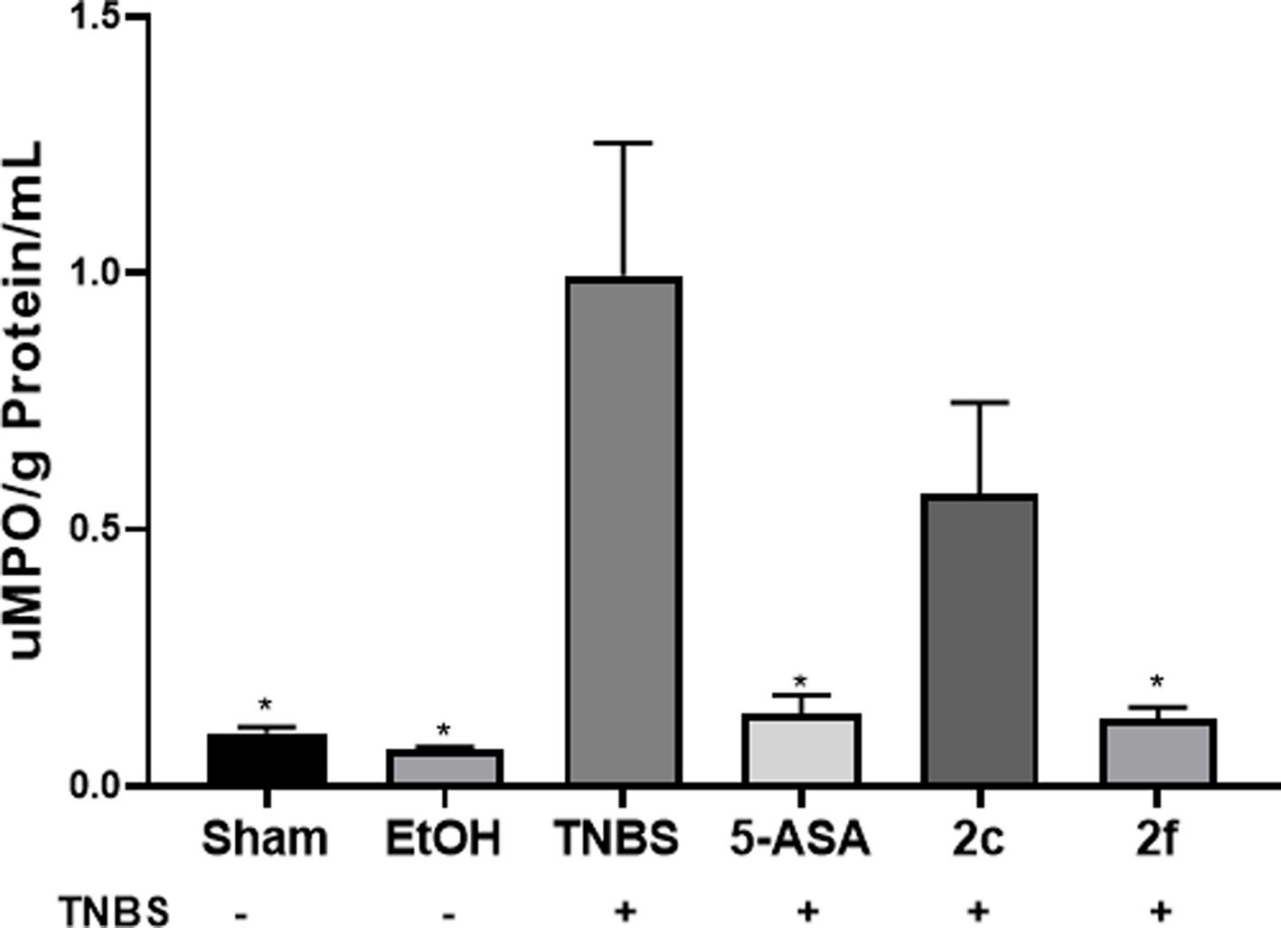
MPO activity in samples from the colon of sham and EtOH rats MPO activity in samples from the colon of sham and EtOH rats and those treated with TNBS, 5-ASA, **2c**, and **2f**. The data are presented as the means ± SEM and were considered significantly different with *P<0.05. * Treatment vs. TNBS group. *n* = 6; assays were performed in triplicate.

The MPO peroxidation activity present in each colonic sample from each rat was quantified. MPO activity indicates neutrophil infiltration in the colon due to the damage caused by TNBS. The obtained data were normalized to the amount of MPO (UMPO) present in 1.0 g of protein and are plotted in Figure 5 versus each treatment.

MPO activity was not significantly different between the sham and EtOH groups. However, a significant difference (*P*<0.05) was observed between the EtOH group and the TNBS group, where the MPO activity increased. In the samples from the groups treated with 5-ASA and **2f**, the MPO activity was significantly lower (*P*<0.05) than that in the samples from the group treated with TNBS. The samples from the group treated with **2c** did not show significant differences from those in the TNBS group.

### Identification of MPO by immunofluorescence

**Figure 6:**
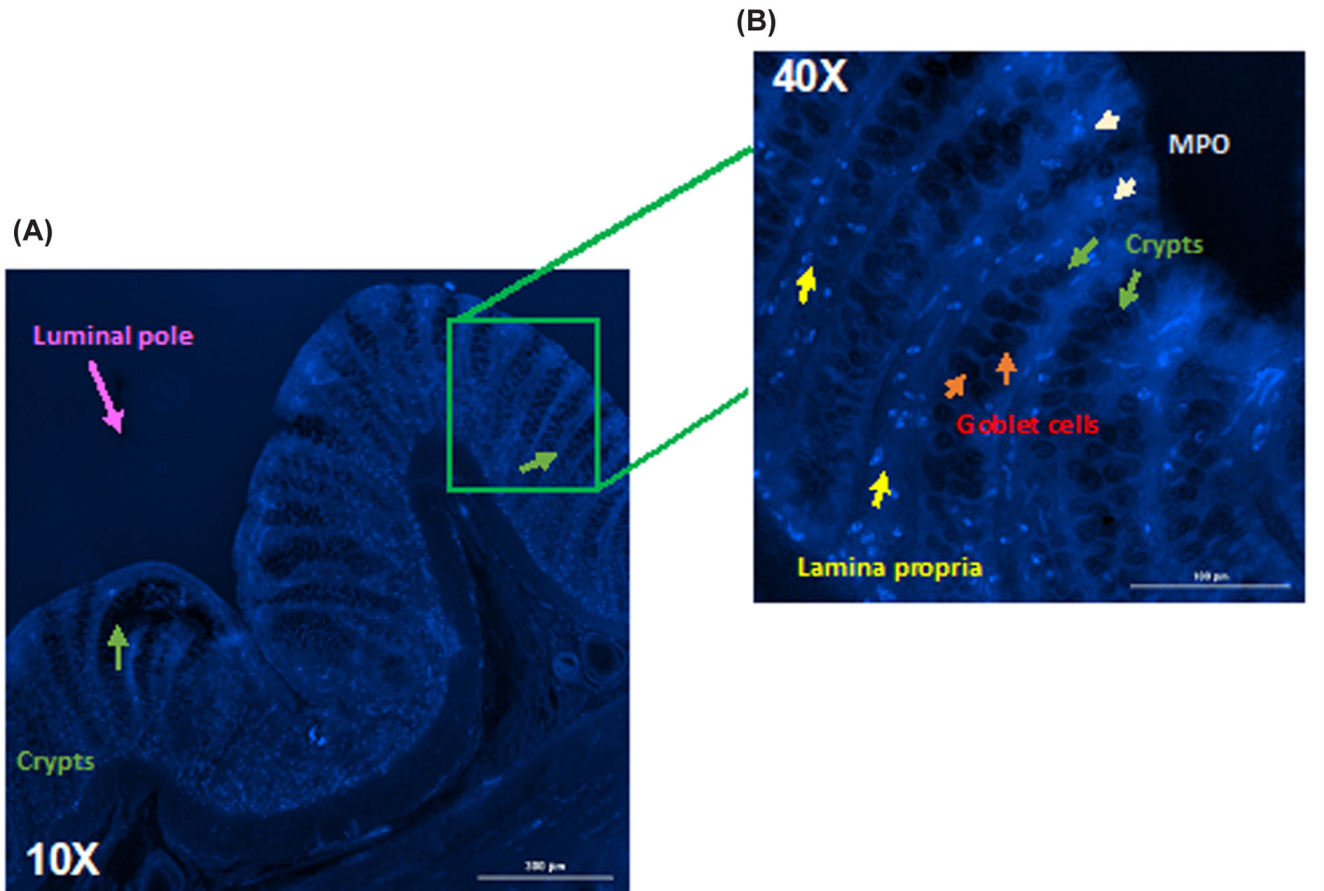
Immunofluorescence of MPO in float sections of the colon sample In (**A**) the colonic mucosa, a significant presence of positive MPO is observed, presenting a tendency toward the luminal pole, lamina propria and base of the crypts (10x), while in (**B**), moderate expression is evident in the lamina propria between the crypts at 40×. (MPO polyclonal antibody, ALEXA FLOUR 350 conjugated, Bioss: MPO is blue)

UC is characterized by inflammation in the mucosa and submucosa; in the mucosa, the crypts and lamina propria are located; these regions were analyzed to determine the presence of MPO due to neutrophil infiltration (Figure 6A). Then, the samples from the EtOH group were analyzed to determine where MPO was located. Figure 6B shows that there was more blue staining due to the presence of MPO between the crypts in the lamina propria. The presence of MPO is observed because this enzyme accumulates at sites of inflammation because it has a greater affinity for some components of the damaged extracellular matrix. Furthermore, biomolecules and fragments damaged by MPO participate in the inflammatory process, resulting in an increasing cycle of adhesion, activity, damage, and altered cellular function. In addition, MPO activation cause tissue damage and dysfunction at sites of leukocyte infiltration [45].

**Figure 7:**
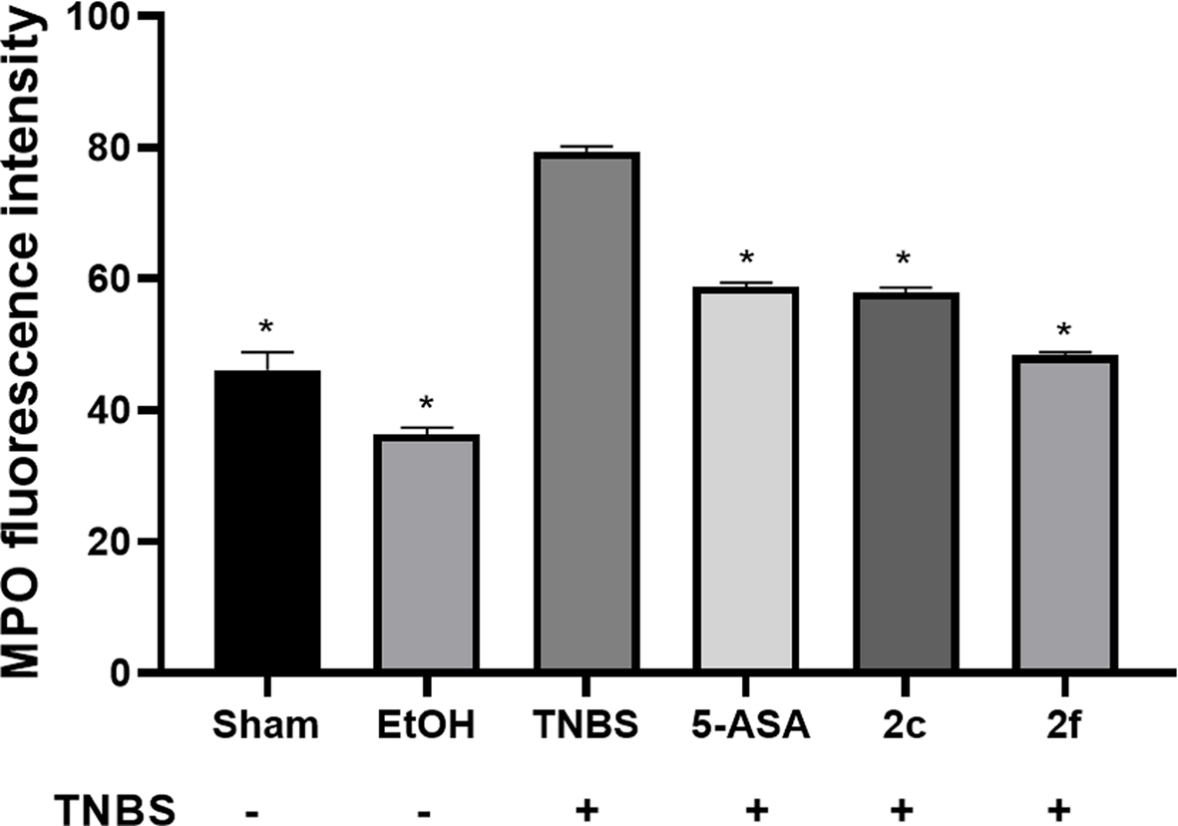
MPO immunofluorescence MPO immunofluorescence in samples from the colon of sham and EtOH-treated rats and those treated with TNBS, 5-ASA, **2c** and **2f**. The MPO fluorescence intensity in float sections of the colon in the different treatment groups was obtained by ImageJ software. The data are presented as the means ± SEMs and were considered to be statistically significant (*P<0.05). n:6 assays were performed in triplicate for the treatment versus TNBS groups.

The fluorescence intensity of the lamina propria region was quantified and plotted, as shown in Figure 7, for the sham, EtOH, TNBS, 5-ASA, **2c** and **2f** groups. The basal MPO concentration in the sham group was measured. Compared with those in the sham and EtOH groups, the levels of MPO in the TNBS group were significantly greater (*P<0.05), as shown in Figure 7. A decrease in the fluorescence intensity was observed in the groups treated with 5-ASA, **2c** and **2f**, indicating the presence of small quantities of stained MPO, which could indicate that Compounds **2c** and **2f** decreased neutrophil infiltration and that less MPO enzyme activity and consequently less inflammation was subsequently detected.

**Figure 8:**
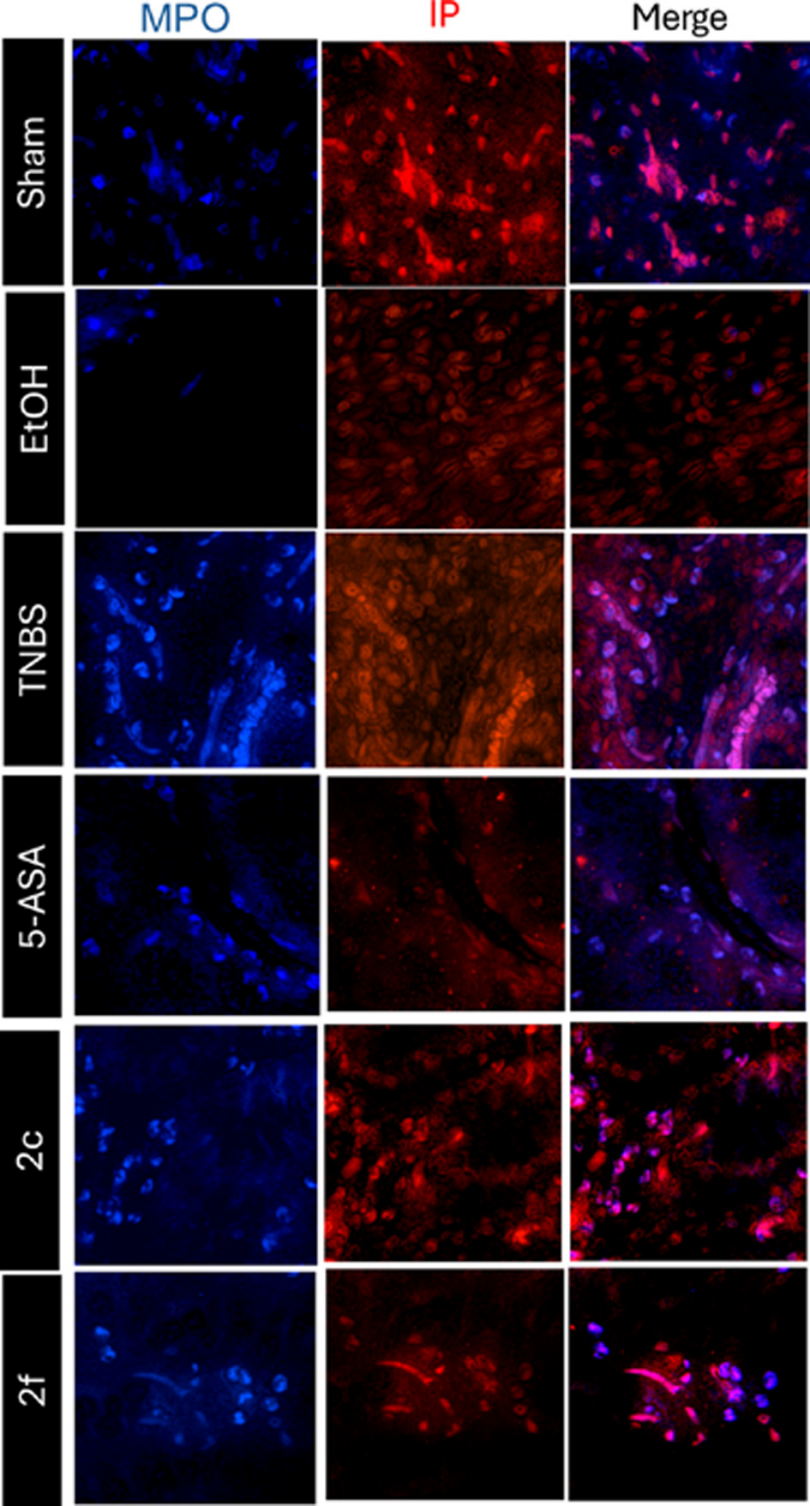
Immunofluorescence of MPO in float sections Immunofluorescence of MPO in float sections of the colon from the different treatment groups. (MPO polyclonal antibody, ALEXA FLOUR 350 conjugated, Bioss: (blue) and propidium iodide for the nucleus of cells (red)). The scale bars represent 100 µm.

Figure 8 shows the MPO immunofluorescence in floating colon sections from the different treatment groups. When correlating the results obtained by immunofluorescence with the quantification of MPO activity using orthodianisidine, it was observed that the sham and EtOH groups presented lower values than did the TNBS group. In the groups treated with Compounds **2c** and **2f**, the MPO levels determined by immunofluorescence assays decreased compared to those in the TNBS group, as in the ortho-dianisidine test.

The presence of MPO might be due to the recruitment of MPO-containing cells (neutrophils/macrophages) due to an inflammatory process. 5-ASA, **2c**, and **2f** decreased cell recruitment, which was reflected by a decrease in the MPO concentration. Although MPO decreased in the **2c** group, this treatment did not effectively inhibit MPO peroxidation activity, as did treatment with 5-ASA and **2f.**

### Lipoperoxidation measurements

**Figure 9:**
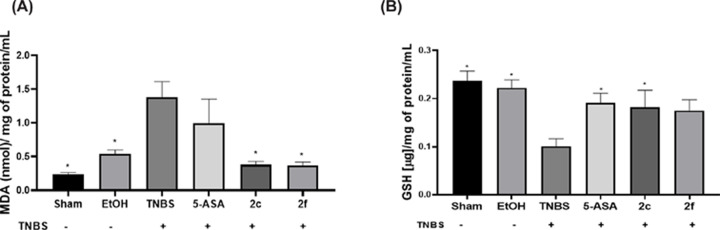
Oxidative stress markers (**A**) Determination of the MDA levels in the colonic tissue of the rats in the treatment and TNBS groups. (**B**) Determination of GSH levels in the colonic tissue of the rats in the treatment and TNBS groups. All assays were performed in triplicate, and statistically significant differences (*P<0.05), n:6, were calculated versus the TNBS group.

MDA levels in each sample group were determined by a lipid peroxidation assay. In the Sham group, the MDA level was considered the basal level. However, a slight increase was observed in the EtOH group due to the ethanol administration. The sham and EtOH groups exhibited significant differences compared with the TNBS group. Furthermore, as shown in Figure 9A, there were significant differences (*P*<0.05) between Groups **2c** and **2f** and the TNBS group. The **2c** and **2f** groups had concentrations close to those of the sham and EtOH groups, indicating that the compounds reduced lipid peroxidation in the UC model and acted as antioxidants even more effectively than did 5-ASA; however, the decrease in the MDA level in the TNBS group was not statistically significant.

### Measurement of reduced glutathione (GSH)

GSH levels were determined in each sample group. The GSH levels in the Sham group were taken as the basal levels. The GSH level in the EtOH group was similar to that in the sham group, but the difference was not significant. However, the GSH level was significantly lower in the TNBS group than in the sham group (*P<0.05**) (**Figure 9B).

Whereas an increase in GSH levels was observed in the 5-ASA and **2c** groups (*P<0.05) compared with the TNBS group, the **2f** group presented an increase in GSH compared with the TNBS group but did not show a significant difference, indicating that they act as antioxidants in the UC model.

## Discussion

UC is characterized by an inflammatory process that involves the infiltration of cells such as neutrophils and macrophages into the intestinal mucosa. These cells contain MPO, among other markers [46] which contribute to increase the reactive oxygen species (ROS). 5-ASA has been used as a reference treatment for mild to moderate UC [47]; therefore, in this work, we focused on evaluating therapeutic options that could contribute to UC treatment by inhibiting MPO and reducing oxidative stress in a UC model induced by TNBS in rat.

The results obtained showed that after TNBS administration, the DAI score confirmed the UC induction as has been reported [8]. After, that the animals were euthanized macroscopic and microscopic studies revealed inflammatory characteristics in the TNBS group. As reported by Antoniou et al. (2016), when TNBS is administered, predominant infiltration of leukocytes and erythrocytes in the mucosa and submucosa occurs after 14 days of administration [8].

Then, the ROS production and consequently the oxidative stress are important hallmarks during UC and the antioxidant molecules has been evaluated such as hydroxycinnamic acid derivatives which are an important class of phenolic acids isolated from plants, such as cinnamic acid, *p*-coumaric acid, ferulic acid, caffeic acid, etc., which have antioxidant and anti-inflammatory activities whose mechanism of action involves the minimization of oxidative damage, acts as signaling molecules able to regulate the expression of antioxidant genes and biochemical pathways [31,48]. Therefore, compounds **2c** and **2f** which are cinnamic derivatives and has antioxidant and MPO inhibitory activity *in vitro* [34] were evaluated as a possible pharmacological treatment for UC.

The microscopic analysis of the colonic tissue samples confirmed that the group induced with TNBS presented neutrophils infiltration which contains MPO and contribute to increase the ROS producing damage on endothelial cells and then epithelial barrier permeability and luminal pathogen invasion. The treatment with 5-ASA and **2f** showed a smaller area of damage in relation to the TNBS group but not the treatment with **2c** [49]. This result agrees with the results from DAI where the compound **2f** present favorable results in treating colitis since the symptoms (diarrhea) diminish.

In addition, the compound **2f** decreases MPO activity, this is a hemoenzyme involved in multiple inflammatory diseases due to produce hypochlorous acid (HOCl) leads to the formation of oxidants and extracellular damage due to MPO is also release from the phagosome to extracellular space resulting in cellular dysfunction and alterations in the gene expression of cell surfaces and the extracellular matrix/glycocalyx [45,50]. Also, HOCl inactivates protease inhibitors and activates collagenolytic metalloproteinases, leading to degradation of the interstitial matrix of the mucosa and epithelial cells [14]. Then, if the compounds **2f**, **2c** and 5-ASA inhibit the MPO activity consequently less HOCl could be produced, which explains the lower degree of tissue damage [51]; due to has been reported by in vitro studies that compounds **2f** and **2c** inhibit the MPO peroxidation and chlorination activities [34]. Then, by inhibiting MPO, the formation of HOCl, which is more oxidizing than superoxide anion and hydrogen peroxide, is prevented [14].

The MPO immunofluorescence analyses confirmed the increased MPO levels in colon samples from rats treated with TNBS. This increase is due to the infiltration of leukocytes caused by an inflammatory process, which leads to an increase in MPO in cells such as neutrophils and macrophages [46,52]. As reported by Dyadyk et al. (2021), a higher concentration of MPO was observed in the lamina propria.

Therefore, the increasing of ROS play a critical role in UC contributing to the progression to colorectal cancer (CRC) [53]. Under oxidative stress condition the ROS produce lipid peroxidation, intestinal mucosal barrier damage, bacterial translocation, and inflammatory response [54,55]. Then, these ROS oxidize unsaturated fatty acids and producing large amount of electrophilic carbonyls (highly reactive α,β-unsaturated), such as malondialdehyde which has been associated with UC [54].The MDA quantification levels in colonic tissue in the TNBS group indicated the increase in lipid peroxidation and consequently the membrane damage which originate more permeability [56,57]. However, the use of **2f** and **2c** compounds decreased MDA inclusive more than 5-ASA.

Glutathione (GSH) is an important molecule for the elimination of intracellular carbonyls by the activity of glutathione-*S*-transferases (GST) who catalyze the conjugation of carbonyls with glutathione. The GSH quantification level in colonic tissue in the TNBS group indicated that it diminishes which could be correlated with the MDA increment during the colitis induction. The compound **2c** and **2f** increasing GSH levels which could be related to hydroxycinnamic acids reduce the lipid peroxidation produced by the hydrogen peroxide (H_2_O_2_) through one electron donation to H_2_O_2_ reducing the cell membrane damage. Also, hydroxycinnamic acids can regulated the oxidative stress production by the heavy metals by free radical scavenging and chelating activities due to heavy metals can be binding to the hydroxyl or carbonyl groups of the hydroxycinnamic acids [58]. Due to these effects hydroxycinnamic acids has been evaluated in different disease such as breast, colon, lung and prostate cancers [59–62]. Furthermore, in the prevention and management of diabetes [63,64]. And in cardiovascular diseases [65,66] between other such as antimicrobial and photoprotective [48,67].

Then, the capacity of **2f** to diminish MDA and increase GSH can be related with its antioxidant activity which was assays previously by DPPH and ABTS [34]. This could be to the presence of OH groups within the aromatic ring, specifically that found in the para-position and it is also present in resveratrol and ferulic acid. However, the **2c** compound has a chlorine group at position 5; its antioxidant activity is lower due to the influence of chlorine since it is an electron-withdrawing group, but in turn, this group favors interaction with MPO, favoring its inhibition. It has been reported that molecules that are used to inhibit MPO peroxidation must also have antioxidant activity since redox-type reactions occur in the MPO cycle [34]. That's why several hydroxycinnamic acids present an anti-inflammatory activity against UC such as cinnamic acid [28], caffeic acid [68,69], and caffeic acid phenethyl ester (CAPE), have been evaluated in models of DSS-induced colitis and showed similar [40], ferulic acid [30].

Therefore, the use of compound **2f** can act as MPO inhibitor and, is able to modulate the ROS production then could modulate NF-*κ*B and p38 MAPK signaling pathways reducing the expression of proinflammatory cytokines and other inflammatory mediators [67,70–73]. NF-κB, is a major modulator of UC, due to this contribute to maintain the intestinal epithelial barrier function and coordinates epithelial immune response to microorganisms in normal intestinal epithelium. NF-κB allow the expression of a variety of proinflammatory cytokines in the intestinal epithelial cells, such as TNF-α, IL-1, IL-8, and COX-2, and promotes inflammation and carcinogenesis [27,74]. Due to during UC, NF-κB expression is increased the inhibition of NF-*κ*B activity is considered practical treatment of UC [75,76]. In addition, the increased ROS production activate extracellular signal-regulated kinases 1 and 2 (Erk1/2), c-Jun N-terminal kinases 1-3 (JNK1-3), or p38 MAPKs which are involved in the UC progression, also changes in p38 MAPK signaling are related with the inflammation level in UC [77,78]. Then, in the future experiments 2f compounds could be evaluated and measure proinflammatory cytokines and some kinases.

## Conclusion

Compound **2f** demonstrated protective effects in an animal model of TNBS-induced UC. After 14 days of treatment, Compound **2f** reduced macroscopic and microscopic colonic damage, the activity and presence of MPO, the concentration of MDA, and the increase in GSH, acting as an antioxidant similar to 5-ASA.

## Supplementary Material

Supplementary Figures S1-S3

## Data Availability

The principal raw data from this manuscript are in the supplementary material. If additional information is required, please get in touch with first or corresponding author to the next email addresses: mrosalesh@ipn.mx, marcrh2002@yahoo.com.mx and astridmriveraa@gmail.com.

## Abbreviations

5-ASA: 5-aminosalicylates
CAPE: caffeic acid phenethyl ester
CRC: colorectal cancer
DAI: disease activity index
DTNB: 5,5'-dithiobis-(2-nitrobenzoic acid)
Erk: extracellular signal-regulated kinase
GSH: glutathione
GST: glutathione-*S*-transferases
IBD: inflammatory bowel disease
MDA: malondialdehyde
MPO: myeloperoxidase
ROS: reactive oxygen species
UC: ulcerative colitis
