# Supplementary material for: Protective effect of (E)-(2,4-dihydroxy)-α-aminocinnamic acid, a hydroxy cinnamic acid derivative, in an ulcerative colitis model induced by TNBS
Source: Biosci Rep. 2024 Oct 4;44(10):BSR20240797. doi: 10.1042/BSR20240797 (PMC11461179; doi:10.1042/BSR20240797)
Supplement: Supplementary Figures S1-S3 [file BSR-2024-0797_supp.pdf]

Supplementary Information

Protective effect of (*E*)-(2,4-dihydroxy)-α-aminocinnamic acid a hydroxy cinnamic acid derivative in an ulcerative colitis model induced by TNBS

Astrid Mayleth Rivera Antonio[1,2], Itzia I. Padilla-Martínez[2], Yazmín K. Márquez-Flores[3], Alan Hipólito Juárez Solano[4,5], Mónica Adriana Torres-Ramos[4,5], , Martha Cecilia Rosales Hernández[1*].

1 Laboratorio de Biofísica y Biocatálisis, Sección de Estudios de Posgrado e Investigación, Escuela Superior de Medicina, Instituto Politécnico Nacional, Plan de San Luis y Salvador Díaz Mirón s/n, Casco de Santo Tomas, Ciudad de México 11340, México.

2 Laboratorio de Química Supramolecular y Nanociencias, Unidad Profesional Interdisciplinaria de Biotecnología, Instituto Politécnico Nacional, Avenida Acueducto s/n, Barrio la Laguna Ticomán, Ciudad de México 07340, México.

3 Departamento de Farmacia, Escuela Nacional de Ciencias Biológicas, Campus Zacatenco, Instituto Politécnico Nacional, Av. Wilfrido Massieu s/n Col. Zacatenco, C.P. 07738, Ciudad de México, México.

4 Dirección de investigación del Instituto Nacional de Neurología y Neurocirugía Manuel Velasco Suárez

***corresponding: marcrh2002@yahoo.com.mx.**

S1. Characterization the compounds **2c** and **2f**

(*E*)-(5-Chloro-2-hydroxy)-α-aminocinnamic acid (**2c**). The synthesis was carried out starting from 0.300 g (1.26 mmol) of **1c** were dissolved in 18 mL of a recently prepared aqueous H_2SO_4 solution of 15% (v/v) inside a 30 mL microwave vial. The reaction was allowed to proceed for 20 min at 160 ◦C and 1200 rpm. Thereafter, the reaction was neutralized with $NaHCO_3$ (0.70 g), and the resulting solid was then filtered and washed with distilled water (10 mL) to yield 0.170 g (0.790 mmol, 63%) of a brown solid, m.p. 252 ◦C, Rf: 0.81 hexane/AcOEt 1:1. ^{1}H-NMR: δ 10.7 (br, 1H, OH), 7.64 (d, 1H, 4J = 2.0, H6), 7.09 (s, 1H, H7), 7.39 (m, 2H, H4, 3). ^{13} C-NMR: δ 158.3 (C=O), 148.1 (C2), 143.0 (C8), 128.9 (C5), 127.6 (C4), 125.8 (C6), 122.8 (C1), 117.9 (C3), 114.1 (C7). IR cm−1: 3381 (N-H), 1720 (C=O), 1518, 919, 835.

(*E*)-(2,4-dihydroxy)-α-aminocinnamic acid (**2f**). The synthesis was carried out starting from 0.300 g (1.26 mmol) of **1f** were dissolved in 18 mL of a recently prepared aqueous H_2SO_4 solution of 5% (v/v) inside a 30 mL microwave vial. The reaction was allowed to proceed for 10 min at 120 ◦C and 1200 rpm. Thereafter, the reaction was neutralized with $NaHCO_3$ (0.70 g), and the resulting solid was then filtered and washed with distilled water (10 mL) to yield 0.180 g (0.920 mmol, 67%) of a brown solid, m.p. 272 ◦C, Rf: 0.77 hexane/AcOEt 1:1. ^{1}H-NMR: δ 10.0 (Br, 2H,

OH), 7.35 (*d*, 1H, 3*J* = 8.5, H6), 7.06 (s, 1H, H7), 6.74 (dd, 1H, 3*J* = 8.5, 4*J* = 2.3, H5), 6.69 (d, 1H, 4*J* = 2.3, H3). ^{13}C NMR: δ 159.2 (C=O), 158.2 (C2), 151.0 (C4), 139.1 (C8), 127.7 (C6), 116.6 (C7), 113.6 (C5), 112.7 (C1), 102.4 (C3). IR cm−1: 3278 (OH, N-H), 1696 (C=O), 1454, 826.

S2. Scheme of reaction. Microwave assisted hydrolysis of acetamidocoumarins **1a-j** to produce (*Z*)-2-hydroxycinnamic acids **2c y 2f**.

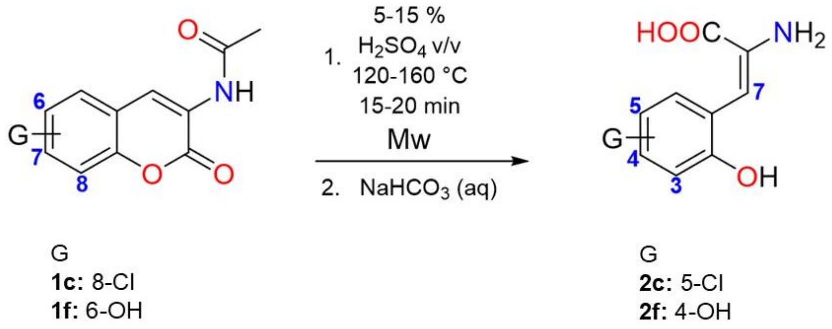

S3. Colonic tissue for macroscopic evaluation

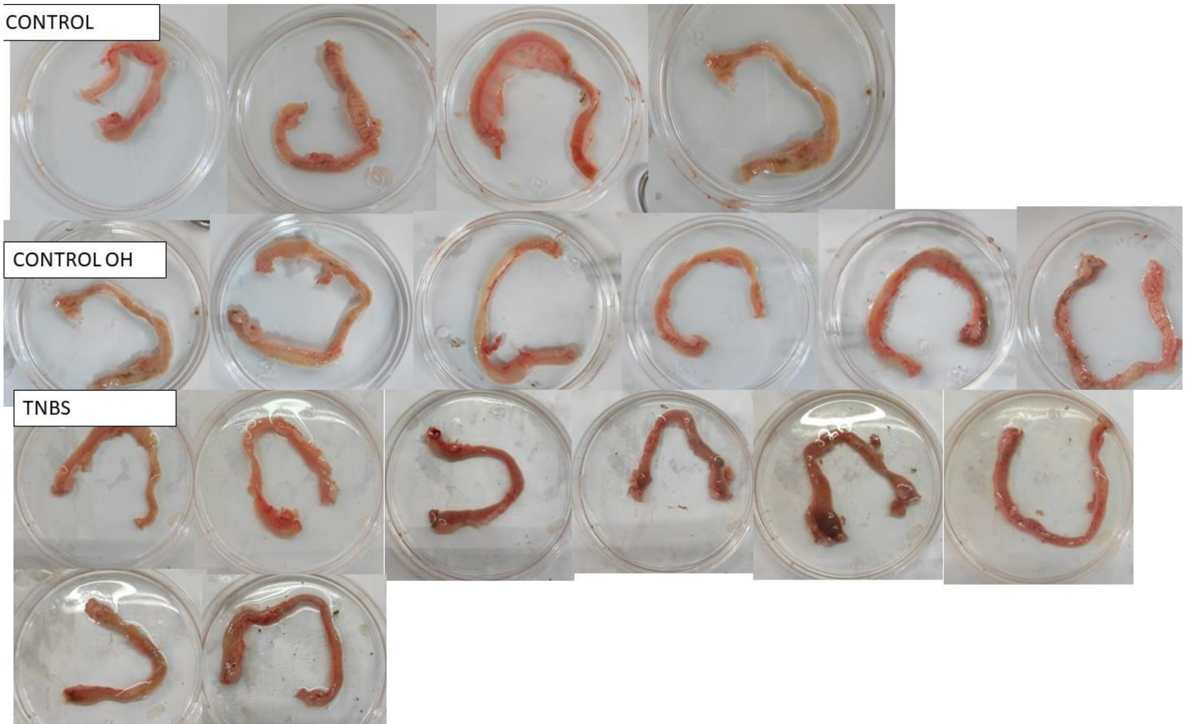

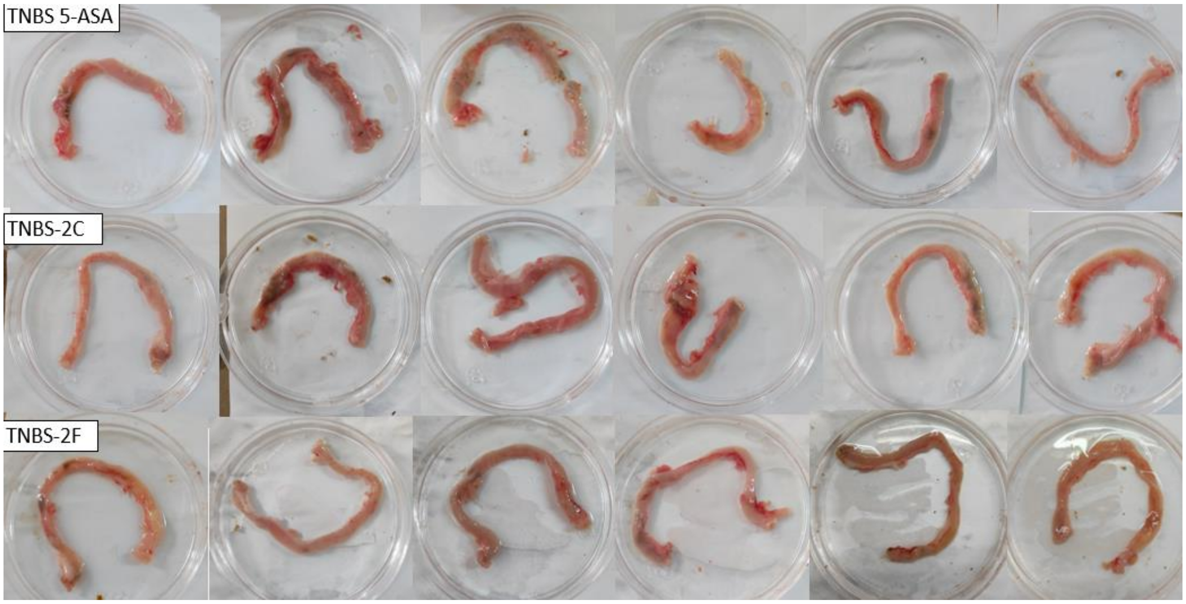

TNBS 5-ASA
TNBS-2C
TNBS-2F
